# Pharmacological potentiators of the calcium signaling cascade identified by high-throughput screening

**DOI:** 10.1093/pnasnexus/pgac288

**Published:** 2022-12-09

**Authors:** Michele Genovese, Daniela Guidone, Martina Buccirossi, Anna Borrelli, Alejandra Rodriguez-Gimeno, Fabio Bertozzi, Tiziano Bandiera, Luis J V Galietta

**Affiliations:** Telethon Institute of Genetics and Medicine (TIGEM), Pozzuoli, 80078 Naples, Italy; Telethon Institute of Genetics and Medicine (TIGEM), Pozzuoli, 80078 Naples, Italy; Telethon Institute of Genetics and Medicine (TIGEM), Pozzuoli, 80078 Naples, Italy; Telethon Institute of Genetics and Medicine (TIGEM), Pozzuoli, 80078 Naples, Italy; D3 PharmaChemistry, Italian Institute of Technology (IIT), Via Morego, 3016163, Genoa, Italy; D3 PharmaChemistry, Italian Institute of Technology (IIT), Via Morego, 3016163, Genoa, Italy; D3 PharmaChemistry, Italian Institute of Technology (IIT), Via Morego, 3016163, Genoa, Italy; Telethon Institute of Genetics and Medicine (TIGEM), Pozzuoli, 80078 Naples, Italy; Department of Translational Medical Sciences (DISMET), Università degli Studi di Napoli “Federico II, C.so Umberto I, 40, 80138 Napoli NA, Italy

**Keywords:** calcium signaling, purinergic receptor, inositol triphosphate receptor, phospholipase C, high throughput screening

## Abstract

Pharmacological modulators of the Ca^2+^ signaling cascade are important research tools and may translate into novel therapeutic strategies for a series of human diseases. We carried out a screening of a maximally diverse chemical library using the Ca^2+^-sensitive Cl^−^ channel TMEM16A as a functional readout. We found compounds that were able to potentiate UTP-dependent TMEM16A activation. Mechanism of action of these compounds was investigated by a panel of assays that looked at intracellular Ca^2+^ mobilization triggered by extracellular agonists or by caged-IP_3_ photolysis, PIP_2_ breakdown by phospholipase C, and ion channel activity on nuclear membrane. One compound appears as a selective potentiator of inositol triphosphate receptor type 1 (ITPR1) with a possible application for some forms of spinocerebellar ataxia. A second compound is instead a potentiator of the P2RY2 purinergic receptor, an activity that could promote fluid secretion in dry eye and chronic obstructive respiratory diseases.

Significance Statement Modulation of the Ca^2+^ signaling pathway with selective pharmacological agents is an important approach to understand the underlying molecular mechanisms and a possible therapeutic strategy for many human diseases. Our study has revealed novel small molecules that act on the pathway at different levels. In particular, they include allosteric potentiators of the purinergic receptor P2RY2 and the inositol triphosphate receptor type 1 (ITPR1), two targets for which there were no available modulators acting with this mechanism. These novel pharmacological agents will allow experiments to selectively modulate the corresponding targets for scientific purposes. They will also be the starting point to develop selective drugs.

## Introduction

The Ca^2+^ signaling cascade is a complex array of molecular mechanisms that transduce extracellular chemical and mechanical stimuli into Ca^2+^ mobilization from intracellular stores or Ca^2+^ influx from the extracellular milieu. Typically, chemical stimuli (hormones, neutrotransmitters, exogenous substances) bind to plasma membrane G protein-coupled receptors (GPCRs) that activate phospholipase C (PLC) enzymes (1). Activated PLCs catalyze the breakdown of phosphatidylinositol bisphosphate (PIP_2_) into inositol 1,4,5-trisphosphate (IP_3_) and diacylglycerol (DAG). IP_3_ binds to receptors (inositol triphosphate receptors, ITPRs) localized in the membrane of the endoplasmic reticulum and working as Ca^2+^-permeable channels (2). There are three types of ITPRs (ITPR1-3) that can form homo-tetramers or hetero-tetramers with different intrinsic characteristics and tissue/subcellular distribution (3). Activation of ITPRs upon IP_3_ binding triggers Ca^2+^ release from the endoplasmic reticulum with local elevation of Ca^2+^ (4) that can in turn modulate the function of a large series of effectors, including other ion channels, Ca^2+^-regulated enzymes, cytoskeleton proteins, and transcription factors (1). An additional mechanism of intracellular Ca^2+^ elevation is influx through plasma membrane proteins belonging to different families: (i) voltage-dependent Ca^2+^ channels; (ii) chemical-, temperature-, and stretch-sensitive TRP channels; and (iii) store-operated ORAI1-3 channels (5).

TMEM16A (official name: ANO1) is a Ca^2+^-activated Cl^−^ channel expressed in different types of tissues and cell types, including airway surface epithelia, exocrine glands, smooth muscle, nociceptive neurons, and olfactory receptors (6–9). TMEM16A is typically activated by purinergic agents (UTP, ATP), histamine, acetylcholine, and other Ca^2+^ agonists through the GPCR-PLC-ITPR cascade. Potentiation of TMEM16A-dependent Cl^−^ transport in airway epithelia could potentially overcome the defect in Cl^−^ secretion that occurs in cystic fibrosis (CF), thus restoring mucociliary clearance (10). TMEM16A-dependent anion transport can be detected in a very sensitive and rapid way with a cell-based assay employing the halide-sensitive yellow fluorescent protein (HS-YFP) (11, 12). For this reason, we decided to use cells co-expressing TMEM16A and HS-YFP in a high-throughput assay to find direct TMEM16A potentiators but also small molecule modulators of Ca^2+^ signaling. Actually, pharmacological modulators of the Ca^2+^ signaling pathway, acting on specific components of the cascade, are important as both research tools and possible therapeutic agents to treat a variety of human diseases. By screening a maximally diverse chemical library, we found compounds acting with specific mechanisms, including potentiation of purinergic receptor P2Y2 (P2RY2) and ITPR1 proteins.

## Results

### Identification of active compounds by high-throughput screening

We used Fischer rat thyroid (FRT) cells that we previously generated by stable transfection with TMEM16A and HS-YFP plasmids (12). FRT cells are a convenient cell model since they do not express other anion channels and transporters. Furthermore, they form tight epithelia with high electrical resistance when plated on porous membrane (13). In this way, transepithelial ion transport in FRT cells can be studied with the short-circuit current technique.

**Fig. 1.**
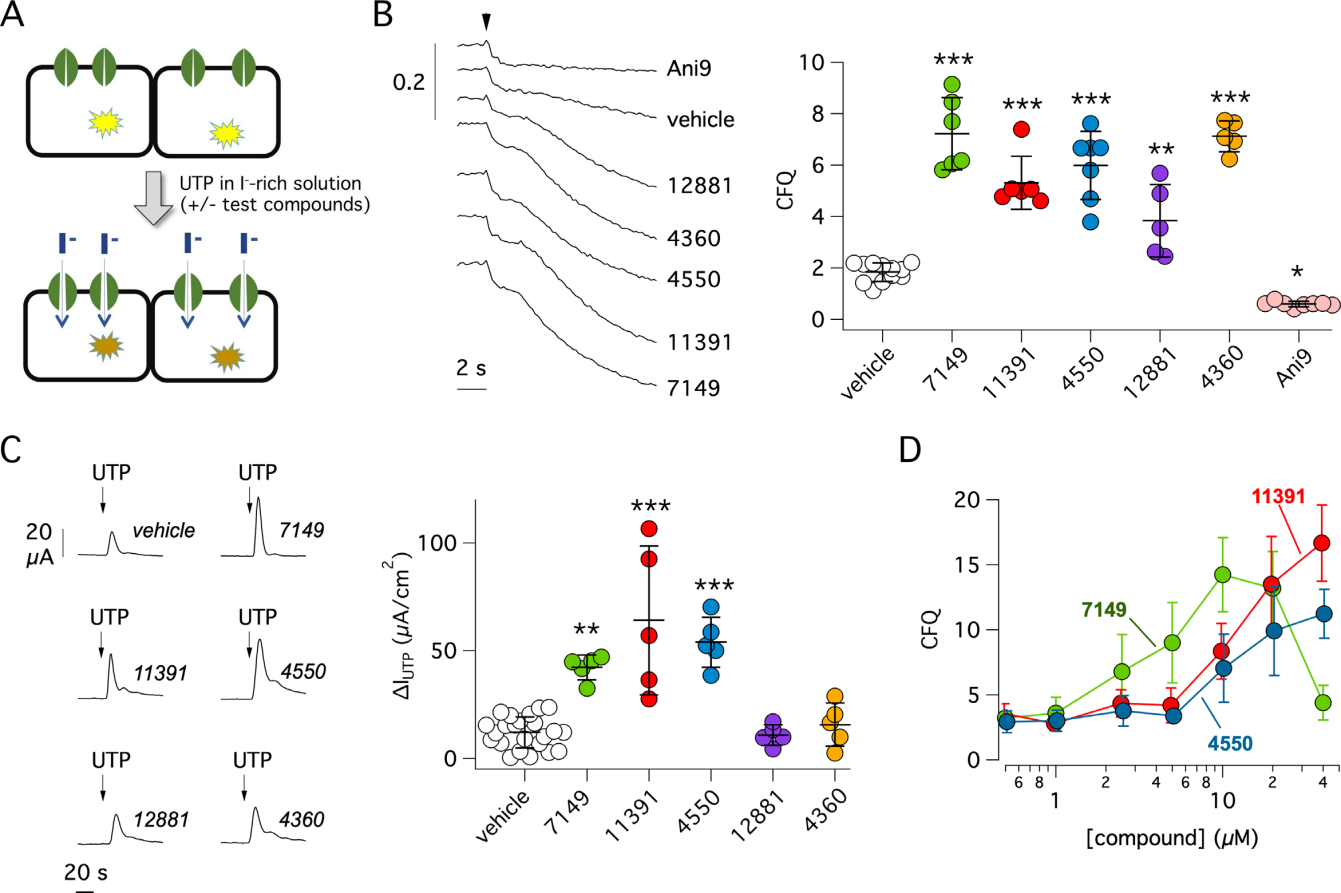
Identification of Ca^2+^ signaling cascade potentiators by high-throughput screening. (A) Scheme of the screening assay. FRT cells with co-expression of the TMEM16A Cl^−^ channel and HS-YFP were preincubated for 20 min with compounds in 96-well microplates. For the assay, the microplate reader continuously recorded cell fluorescence before and after addition of a saline solution containing I^−^ instead of Cl^−^ plus a submaximal UTP concentration (0.25 µM). TMEM16A channel activation by UTP resulted in I^−^ influx and HS-YFP quenching. Presence of an active compound in the well was detected by faster and larger quenching. (B) Detection of active compounds by HS-YFP assay. Representative traces (left) and summary of data (right) obtained for indicated compounds. The scatter dot plot reports activity as cumulative fluorescence quenching (CFQ). *P <0.05; **P <0.01; ***P <0.001 vs. vehicle (ANOVA with Dunnett’s post-hoc test). (C) Representative traces (left) and summary of data (right) from short-circuit current (Isc) recordings on FRT cells with stable expression of TMEM16A. Cells were briefly pre-incubated with indicated compounds (10 µM) or vehicle and then stimulated with 0.25 µM UTP (on the apical side) to induce TMEM16A-dependent Cl^−^ transport. The scatter dot plot reports the value of maximal UTP effect. The current activated by UTP is significantly enhanced by ARN7149, ARN11391, and ARN4550 compared to vehicle. **P <0.01; ***P <0.001 (ANOVA with Dunnett’s post-hoc test). (D) Dose-response of ARN7149, ARN11391 and ARN4550 by HS-YFP assay in FRT cells expressing TMEM16A.

For the screening, FRT cells were plated at high density in 96-well microplates. After 48 h, TMEM16A activity was evaluated in a microplate reader by injection in each well of a saline solution containing I^−^ instead of Cl^−^ and a submaximal (0.25 µM) UTP concentration (Fig. 1A). Upon injection, UTP triggers Ca^2+^ mobilization and TMEM16A activation. The resulting TMEM16A-dependent I^−^ influx causes HS-YFP quenching. The presence in the well of a small molecule that potentiates TMEM16A activity, in a direct or indirect way, is therefore expected to enhance the rate of HS-YFP quenching.

We screened a chemical library of 11,300 compounds, generated at the Italian Institute of Technology, and having maximal structural diversity and good drug-like properties. This library was previously used to find CFTR correctors and potentiators (14, 15). All compounds were tested at 10 µM by addition in the microplate 20 min before the assay. Control wells in each microplate included vehicle alone or TMEM16A inhibitor Ani9 (16). Primary hits, showing potentiation of TMEM16A activity, were retested in the HS-YFP assay to confirm activity (Fig. 1B). Compounds passing this test were further evaluated in short-circuit current recordings on FRT epithelia. As shown in Fig. 1C, the peak of Cl^−^ current elicited by UTP was significantly enhanced by three compounds: ARN7149, ARN11391, and ARN4550. The lack of effect of other two compounds, ARN12881 and ARN4360, in the short-circuit current assay could imply that they act on an endogenous electroneutral anion transporter and not on TMEM16A. We decided to continue the characterization of ARN7149, ARN11391, and ARN4550. We determined the dose-response relationships for these three compounds testing multiple concentrations with the HS-YFP assay (Fig. 1D). ARN11391 and ARN4550 were effective at concentrations ≥10 µM. ARN7149 had instead a bell-shaped dose-response relationship with activity in the 5 to 20 µM range.

### Mechanism of action of active compounds on intracellular Ca^2+^ mobilization

**Fig. 2.**
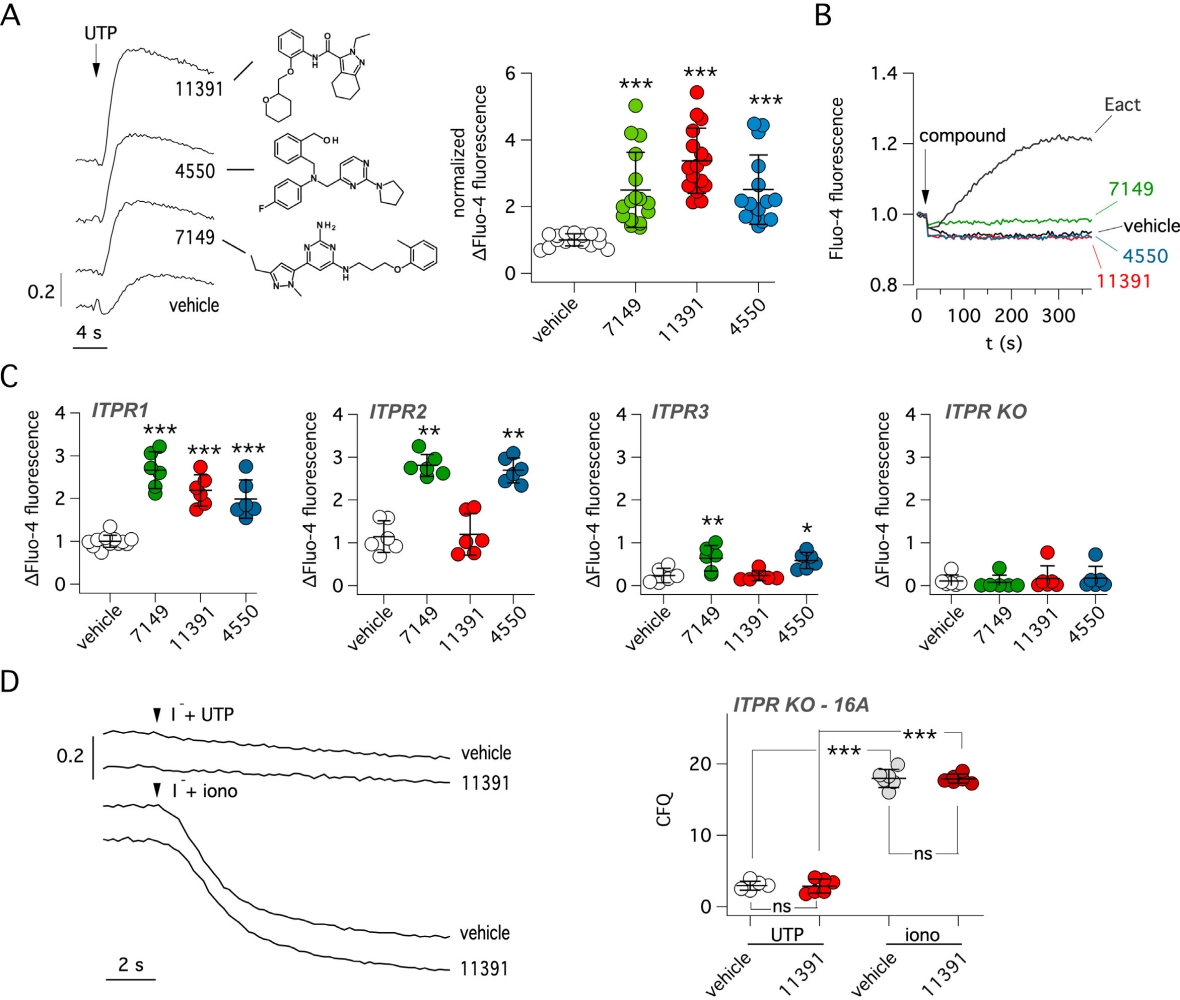
Effect of active compounds on Ca^2+^ mobilization. (A) Left: representative traces showing effect of 0.25 µM UTP (with/without indicated compounds, 10 µM) on Fluo-4 fluorescence in null FRT cells. The chemical structures of compounds are shown. Right: summary of UTP effect on Fluo-4 fluorescence. The symbols report the normalized maximal change in fluorescence caused by UTP with vehicle or compounds (10 µM). ***, *P* < 0.001 vs. vehicle (ANOVA with Dunnett's post-hoc test). (B) Representative traces showing the time-course of Fluo-4 fluorescence following acute addition (arrow) of vehicle, indicated compounds from the screening (10 µM), or Eact (5 µM) as a TRPV4 agonist. (C) Summary of data obtained with the Fluo-4 assay in HEK293 cells with selective expression of ITPR1, ITPR2, ITPR3 or totally devoid of ITPR expression (ITPR KO). Ca^2+^ elevation was induced by UTP. *P <0.05; **P <0.01; ***P <0.001 vs. vehicle (ANOVA with Dunnett’s post-hoc test). (D) Representative traces (left) and summary of data (right) from HS-YFP assay carried out in ITPR-defective HEK293 cells transiently transfected with TMEM16A. Cells were stimulated with 5 µM UTP or 1 µM ionomycin (iono) with/without 10 µM ARN11391. ***P <0.001 (ANOVA with Tukey’s post-hoc test).

We monitored intracellular Ca^2+^ mobilization with the Fluo-4 Ca^2+^-sensitive fluorescent probe. For these experiments, we used parental FRT cells devoid of TMEM16A expression. Figure 2A shows that ARN7149, ARN11391, and ARN4550 were all able to potentiate UTP-dependent Ca^2+^ increase. These results indicated that the three compounds act on TMEM16A with an indirect mechanism of action. Subsequently, we determined whether these compounds were able to directly mobilize Ca^2+^ in the absence of UTP. Compounds were injected during recording of Fluo-4 fluorescence (Fig. 2B). We found no effect in contrast to Eact, a compound that elicits Ca^2+^ influx by activating TRPV4 channel (17). These results indicated that ARN7149, ARN11391, and ARN4550 act as potentiators on components of the Ca^2+^ signaling cascade and not Ca^2+^-elevating agents by themselves.

To elucidate the mechanism of action of active compounds identified in the screening, we carried out a series of experiments. Since, Ca^2+^ release mediated by ITPR opening is a key step in the Ca^2+^ signaling cascade, the first set of experiments was done on HEK293 cells in which selective ablation of endogenous ITPR genes were obtained by gene editing (18, 19). Ca^2+^ mobilization elicited by UTP was evaluated with the Fluo-4 probe (Fig. 2C). Interestingly, ARN7149 and ARN4550 were always effective in potentiating the Ca^2+^ increase irrespective of ITPR type expression. In contrast, ARN11391 was only effective in cells with ITPR1 as the only ITPR type (Fig. 2C). As expected, no Ca^2+^ mobilization by UTP ± potentiator was observed in HEK293 cells completely devoid of ITPRs (Fig. 2C). The lack of effect in ITPR-defective cells was also investigated using TMEM16A as a reporter. For this purpose, HEK293 cells without ITPR1-3 expression were transiently transfected with plasmids coding for TMEM16A and HS-YFP. In agreement with the lack of Ca^2+^ mobilization, the HS-YFP assay showed no activation of TMEM16A by UTP with/without ARN11391 (Fig. 2D), Instead, a large effect was observed when Ca^2+^ was directly increased by ionomycin (Fig. 2D).

**Fig. 3.**
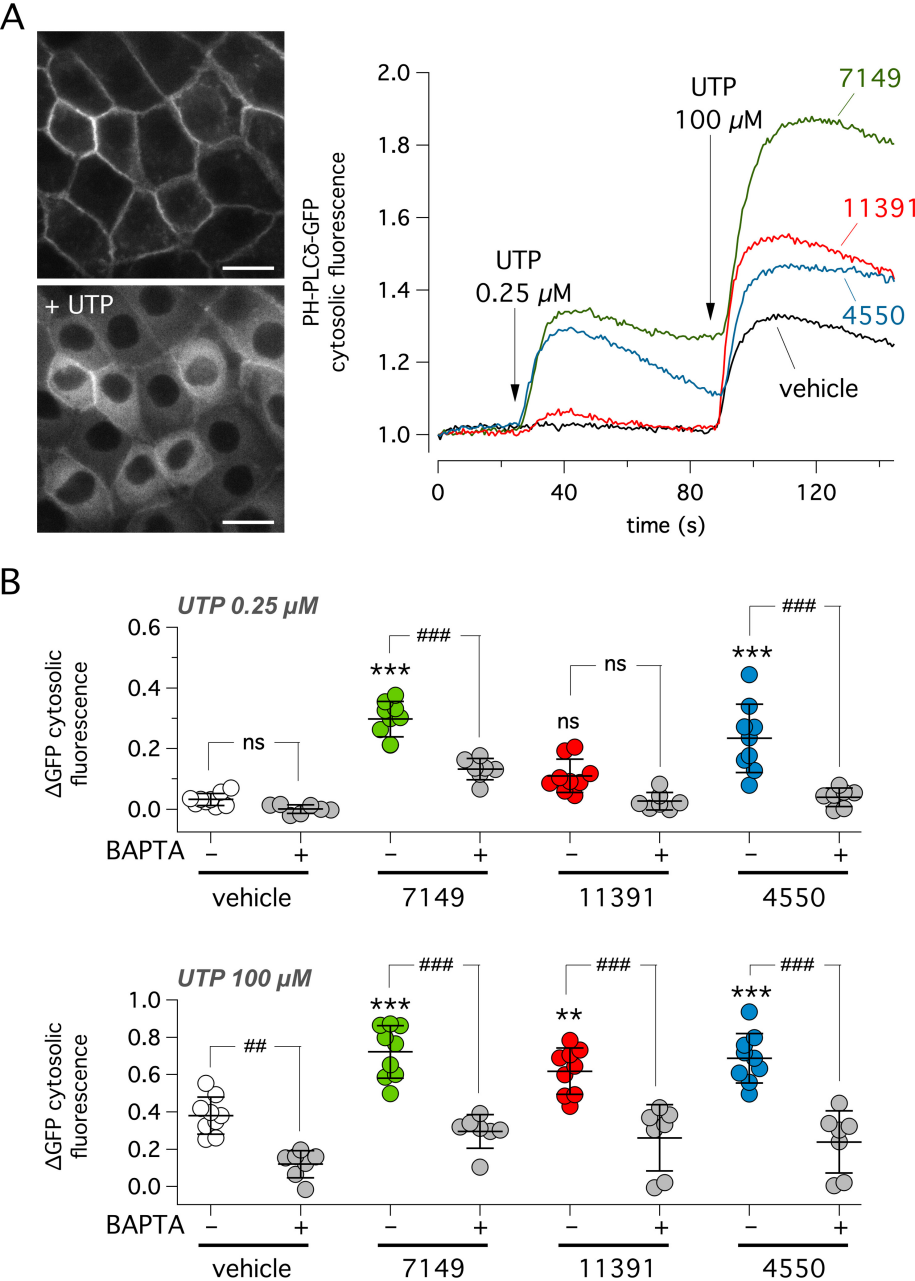
Effect of active compounds on PLC activity. (A) Representative images (left) and GFP fluorescence traces (right) showing relative changes in cytosolic GFP fluorescence in FRT cells stably expressing PH-PLCδ-GFP probe. During the assay, cells were sequentially stimulated with low (0.25 µM) and high (100 µM) UTP concentration. PLC activation, causing PIP_2_ breakdown, results in detachment of the probe from the plasma membrane and redistribution to the cytosol. Vehicle or indicated compounds (10 µM) were added to the cells 20 min before the assay. (B) Summary of data showing the increase in cytosolic PH-PLCδ-GFP localization elicited by the addition of UTP (0.25 µM, top; 100 µM, bottom) in presence of vehicle or compounds. Where indicated, cells were pre-incubated with the membrane-permeable BAPTA/AM to chelate cytosolic Ca^2+^. ***P <0.001 vs. vehicle without BAPTA. ##*P* <0.01; ###*P* <0.001 vs. indicated condition. ns: not significant (ANOVA with Tukey’s post-hoc test).

The second type of experiments was done with an assay that we developed to monitor PLC activity. We generated FRT cells with stable expression of the PH-PLCδ-GFP fluorescent sensor. Under resting conditions, the pleckstrin homology (PH) domain, which binds to PIP_2_, anchors the sensor to the inner side of the membrane (Fig. 3A). Upon activation of PLC by UTP, PIP_2_ hydrolysis releases PH-PLCδ-GFP, which then redistributes to the cytosol. We sequentially added UTP at two concentrations (0.25 and 100 µM), in the presence/absence of potentiators, and measured the increase in GFP fluorescence in the cytosol as the parameter reflecting PLC activation (Fig. 3A). With the lower UTP concentration, ARN7149 and ARN4550, but not ARN11391, significantly potentiated UTP effect. With the higher UTP concentration, all three compounds were effective. It is known that phospholipase C is a Ca^2+^-activated enzyme. Consequently, PLC activity can be amplified through a positive feedback loop based on Ca^2+^ release through ITPRs (20). For this reason, we carried out parallel experiments with the membrane-permeable Ca^2+^ chelant BAPTA/AM. With this compound, PLC activity, with/without compounds, was markedly inhibited (Fig. 3B).

**Fig. 4.**
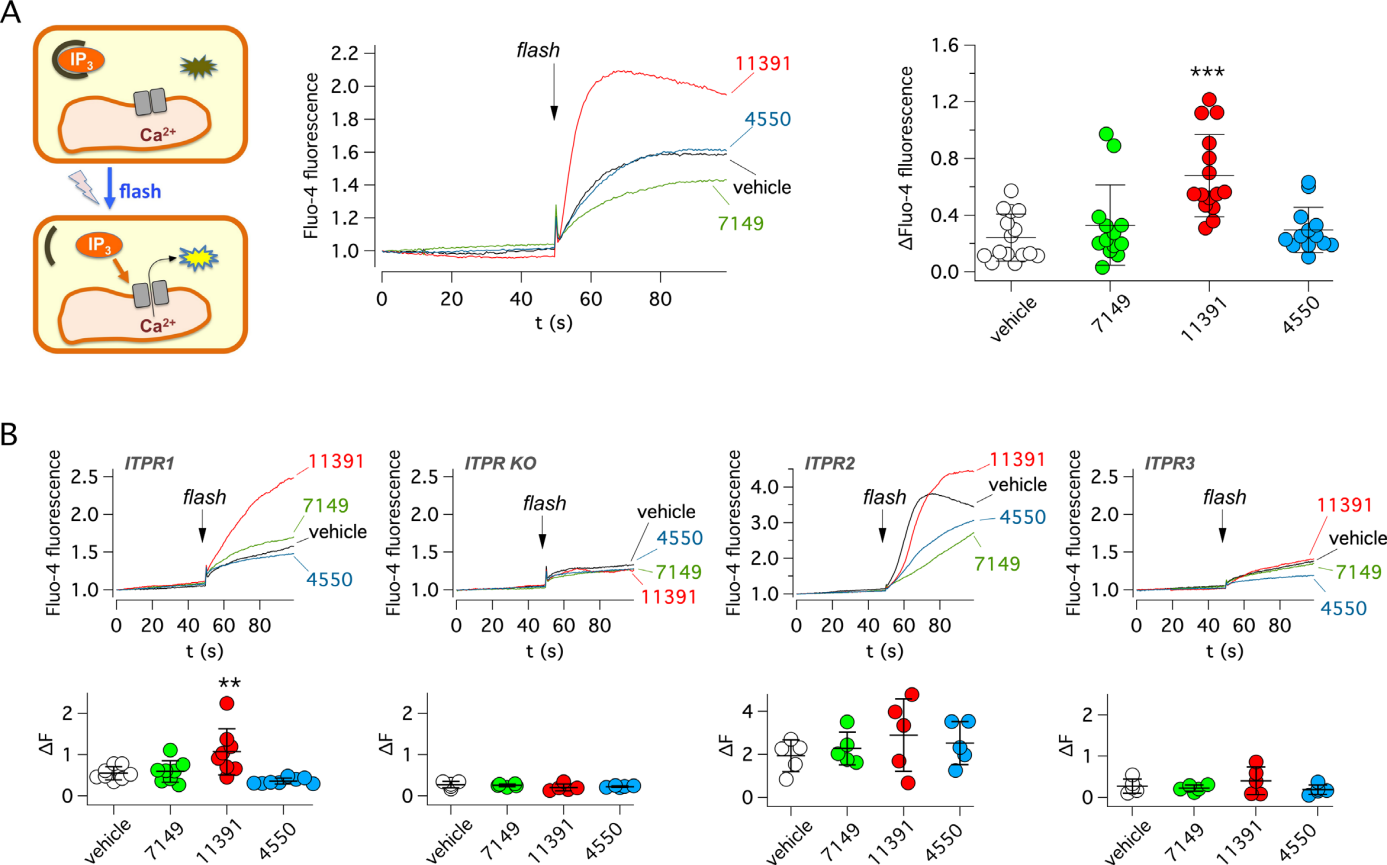
Evaluation of active compounds on ITPR function. (A) Left: scheme of IP_3_ uncaging experiments. Cells were loaded with ci-IP_3_/PM and Fluo-4. Ca^2+^ release from IP3 sensitive stores was elicited with a light flash. Middle: representative traces from experiments on FRT cells showing intracellular Ca^2+^ increase by ci-IP_3_ photolysis. Experiments were done in the presence of vehicle or indicated compounds (10 µM). Right: summary of data. Each symbol shows the maximal amplitude of Fluo-4 increase for indicated conditions. ***P <0.001 vs. vehicle (ANOVA with Dunnett's post-hoc test). (B) IP_3_ uncaging experiments in HEK293 cells with expression of a specific ITPR or totally devoid of ITPR expression (ITPR KO). Top: representative traces. Bottom: summary of data. Each symbol shows the maximal amplitude of Fluo-4 increase for indicated conditions. **P <0.01 vs. vehicle (ANOVA with Dunnett’s post-hoc test).

Based on results shown in Fig. 2C, we hypothesized that ARN11391 directly acts on ITPR1. Therefore, we carried out experiments with a membrane-permeable caged-IP_3_ (ciIP_3_/AM). Upon cell loading, an intense flash of light was applied to cause photolysis and release of free IP_3_ (Fig. 4A). In this way, Ca^2+^ mobilization could be induced by directly activating ITPRs thus, bypassing purinergic receptors and PLC. With this assay, done in FRT cells, only ARN11391 showed activity (Fig. 4A). The caged-IP_3_ experiments were also carried out in HEK293 cells with selective expression of ITPR1 (HEKR1), IPTR2 (HEKR2), ITPR3 (HEKR3), or no ITPR at all (HEK3XKO). Of the three potentiators, ARN11391 was uniquely effective in cells with selective expression of ITPR1 (Fig. 4B).

**Fig. 5.**
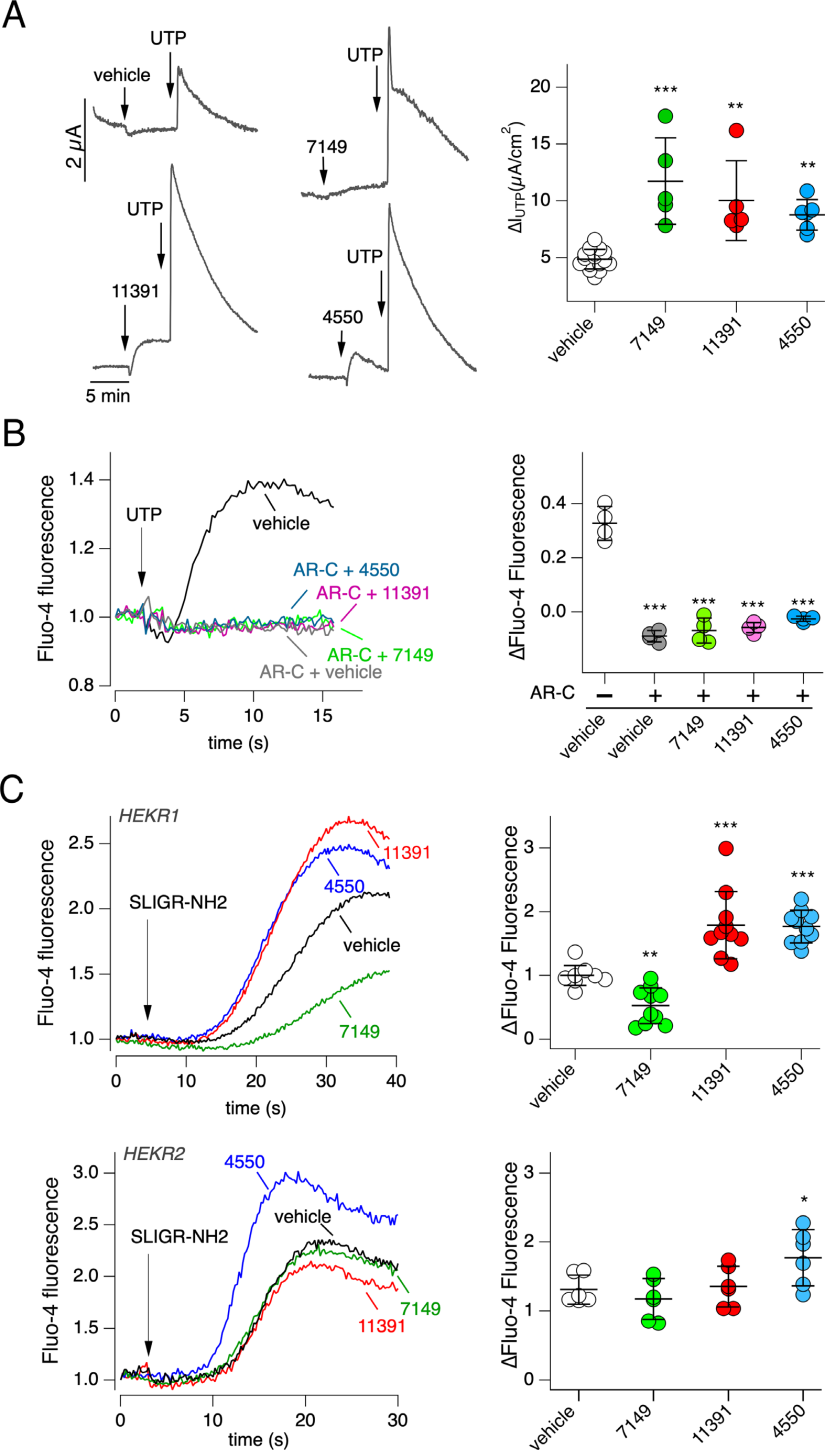
Mechanism of action of ARN7149. (A) Representative traces (left) and summary of data (right) from short-circuit current recordings on human cultured bronchial epithelia. Ca^2+^-dependent Cl^−^ secretion mediated by TMEM16A was triggered with 0.25 µM UTP on the apical side, in the presence of vehicle or indicated compounds: ARN7149 (10 µM), ARN11391 (20 µM), or ARN4550 (20 µM). To avoid the confounding effect of other channels, recordings were done in the presence of: amiloride (10 µM) to block ENaC; paxillin (10 µM) to block large conductance Ca^2+^-dependent K^+^ channels; inh-172 (10 µM, apical) to block CFTR. **P <0.01; ***P <0.001 vs. vehicle (ANOVA with Dunnett’s post-hoc test). (B) Role of P2RY2 in mediating the effect of UTP on Ca^2+^ mobilization. Left: representative traces showing Fluo-4 fluorescence time-course in null FRT cells following extracellular addition of UTP (0.25 µM). Cells were preincubated with/without AR-C118925XX (10 µM) antagonist ± ARN7149, ARN11391, or ARN4550. Right: summary of data. The symbols report the maximal change in Fluo-4 fluorescence. ***P <0.001 vs. experiments without AR-C118925XX (ANOVA with Dunnett’s post-hoc test). (C) Effect of compounds on Ca^2+^ mobilization triggered by SLIGR-NH2 (protease-activate receptor agonist) in HEKR1 (top) and HEKR2 (bottom) cells. Left: representative traces showing Fluo-4 fluorescence time-course following extracellular addition of SLIGR-NH2 (10 µM for HEKR1 and 4 µM for HEKR2). Cells were preincubated with vehicle or indicated compounds (10 µM). Right: summary of data. The symbols report the maximal change in fluorescence. *P <0.05; **P <0.01; ***P <0.001 vs. vehicle (ANOVA with Dunnett’s post-hoc test).

To further evaluate the activity of potentiators on purinergic signaling, we tested them in native airway epithelia, in which UTP is able to activate Ca^2+^-dependent Cl^−^ secretion (21, 22). Figure 5A shows that the transepithelial Cl^−^ current elicited by apical UTP application was significantly enhanced by ARN7149, ARN11391, and ARN4550.

We asked whether the potentiation of UTP stimulus involves P2RY2 or another type of purinergic receptor. Therefore, we used AR-C118925XX as a selective P2RY2 antagonist (23). This compound completely blocked the effect of UTP alone as well that of UTP plus potentiators (Fig. 5B, compare with Fig. 2A).

We also tested the activity of potentiators on other types of stimuli inducing GPCR-PLC-ITPR cascade. Therefore, instead of UTP, we used SLIGR-NH2 that is an agonist of protease-activated receptors (24). These experiments were done on HEK293 cells expressing ITPR1 (HEKR1) or ITPR2 (HEKR2). Interestingly, ARN11391 and ARN4550, but not ARN7149, were effective as potentiators in HEKR1 cells (Fig. 5C, top). Actually, ARN7149 caused a significant inhibition in this set of experiments. In HEKR2 cells, ARN4550 was the only compound that potentiated the SLIGR-NH2 stimulus (Fig. 5C, bottom).

### ARN11391 as an ITPR1 potentiator

**Fig. 6.**
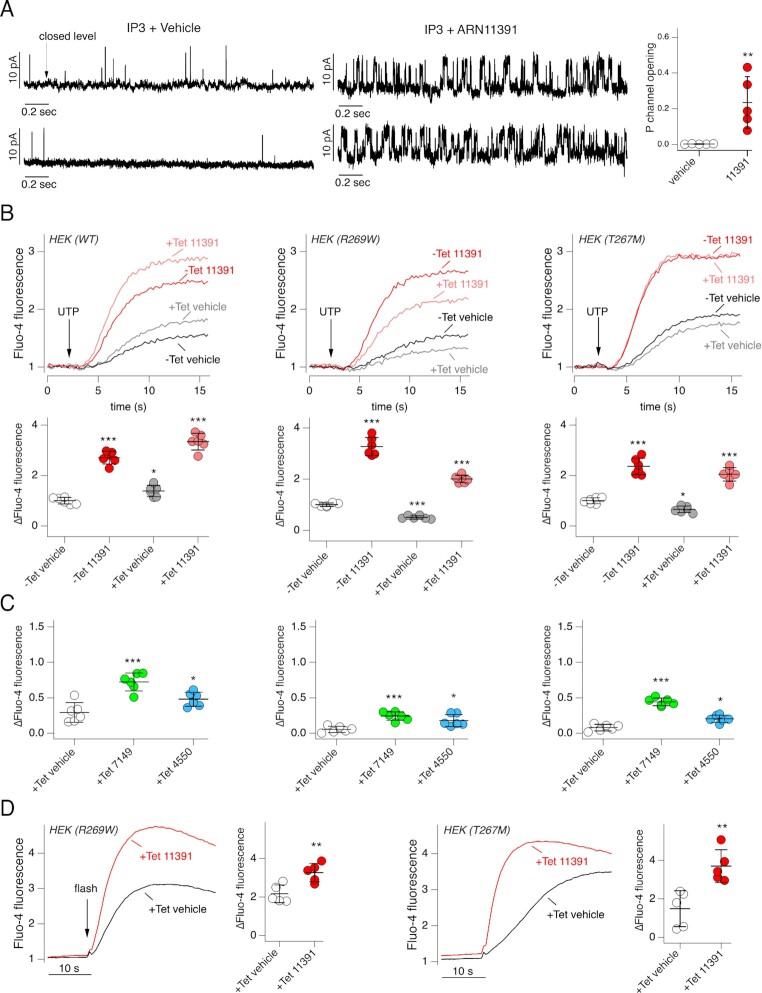
Mechanism of action of ARN11391. (A) Effect of ARN11391 on ITPR1 channel activity. Left: representative single-channel currents recorded in on-nucleus patch-clamp experiments from HEK293 (HEKR1) cells stably expressing ITPR1-YFP (V*p* = + 40-mV). Each trace is from a separate experiment. Right: open channel probability for experiments with vehicle or ARN11391 (20 µM) in the pipette solution. **P <0.01 vs. vehicle (Student’s *t-*test). (B) Effect of ARN11391 on HEK293 cells with inducible (tetracycline) expression of wild type or mutant ITPR1. Top: representative traces showing changes in Fluo-4 fluorescence elicited by UTP (5 µM). Cells were preincubated with vehicle (DMSO) or ARN11391 (10 µM). Where indicated, tetracycline (Tet) was added to cells to induce IPTR1 expression. Bottom: summary of data. The symbols report the maximal change in fluorescence. *P <0.05; **P <0.01. ***P <0.001 vs. vehicle (ANOVA with Dunnett’s post-hoc test). (C) Data from Fluo-4 experiments done on HEK293 cells expressing wild type (left), R269W (middle), and T267M (right) ITPR1. Cell were previously treated with tetracycline and then stimulated with 5 µM UTP as in (B). *P <0.05; ***P <0.001 (ANOVA with Dunnett's post-hoc test). (D) IP3 uncaging experiments carried out in HEK293 cells expressing the indicated mutant ITPR1. Cells were treated with tetracycline before experiments. Uncaging was done in the presence and absence of 10 µM ARN11391. **P <0.01 (Student’s *t*-test).

We were particularly intrigued by the possibility of ARN11391 being a direct ITPR1 potentiator. Actually, to the best of our knowledge, there are no known selective activators/potentiators of ITPRs in general. Therefore, we used cells overexpressing ITPR1 to carry out nuclear patch-clamp recordings (25, 26). In these experiments, the outer nuclear membrane serves as a surrogate of endoplasmic reticulum thus allowing recording of ITPR single channel activity (25, 26). Experiments were done with/without ARN11391 in the pipette solutions (Fig. 6A). We observed single channel openings of the expected current amplitude for ITPR1 (∼10 pA at + 40 mV) in 5 out of 40 attempts with vehicle alone, and in 5 out of 32 attempts with ARN11391. In the absence of compound (vehicle alone), openings were rare and open channel probability (Po) was well below 0.01. With ARN11391, recordings showed much higher channel activity, with an average Po value close to 0.2 (Fig. 6A).

ITPR1 gene may be affected by loss-of-function mutations in some forms of spinocerebellar ataxia (SCA29) (27). We asked whether ARN11391 is effective in cells expressing mutant ITPR1. We tested the compound on two SCA29 mutations: R269W and T267M. Experiments were done on cells with inducible expression of these ITPR1 mutants (28). We pretreated cells with/without tetracycline and then recorded the intracellular Ca^2+^ increase elicited by UTP. Results were compared with those of cells expressing wild type ITPR1. Induction had opposite effects depending on the cell type: an increase in signal for wild type ITPR1 and a significant decrease for R269W- and T267M-ITPR1 (Fig. 6B). We interpret this decrease as the dominant negative effect caused by overexpression of mutant ITPR1 over endogenous ITPRs. Importantly, ARN11391 elicited a marked potentiation of Ca^2+^ mobilization in cells with R269W and T267M mutants (Fig. 6B). For comparison, we also tested ARN7149 and ARN4550 in cells expressing mutant ITPR1 (Fig. 6C). These compounds were also effective although less than ARN11391.

The efficacy of ARN11391 on mutant ITPR1 was also evaluated with the caged IP_3_ assay. In cells expressing R269W- and T267M-ITPR1, ARN11391 significantly amplified the Ca^2+^ mobilization elicited by IP3 uncaging (Fig. 6D).

## Discussion

In our study, we used the activity of the Ca^2+^-activated TMEM16A Cl^−^ channel as the functional readout to identify TMEM16A potentiators as well as modulators of the Ca^2+^ signaling cascade. After the primary screening of a chemical library and secondary tests, we found three compounds, ARN7149, ARN11391, and ARN4450, that significantly potentiated the effect of UTP on TMEM16A. We applied a series of functional assays to assess the mechanism of action of compounds. For two of them, ARN11391 and ARN7149, we found convincing evidence indicating ITPR1 and P2RY2 as the probable targets, respectively.

Regarding ARN11391, we found that it is the only compound effective in the caged IP_3_ assay in which all other upstream steps (membrane receptors, PLC) are bypassed. Furthermore, ARN11391 is only effective when ITPR1 is expressed. We also carried out nuclear patch-clamp experiments on ITPR1-expressing cells that demonstrated a marked increase in channel activity when ARN11391 was included (Fig. 6A). Such results are supportive of a mechanism involving direct interaction of the compound with ITPR1 protein. Intriguingly, in the PLC assay, we found that ARN11391 was ineffective with the submaximal UTP stimulus, but effective with the maximal UTP stimulus. This latter result could appear inconsistent with a mechanism based on ITPR1 binding since PLC is localized upstream in the signaling cascade. However, we think that the effect of ARN11391 on PLC can be explained with a positive feedback loop connecting ITPR activation and Ca^2+^-dependent PLC (20). Indeed, this link is demonstrated by showing that PLC activity can be markedly inhibited with the BAPTA Ca^2+^-chelating agent. Interestingly, ARN11391 was effective in cells expressing ITPR1 with mutations causing SCA29. Such results may indicate a possible therapeutic application of ARN11391-like compounds in patients with spinocerebellar ataxia caused by ITPR1 defective function. We noticed that ARN7149 and ARN4550 showed some activity on ITPR1 mutants (Fig. 6C). In this respect, R269W and T267M have been shown to decrease affinity of ITPR1 for IP_3_ (28), probably by affecting the IP_3_ binding site (27). Therefore, the effect of ARN7149 and ARN4550 can be explained with an indirect mechanism on ITPR1, due to enhanced IP_3_ concentration that overcomes the decreased affinity of the mutant receptor for IP_3_.

ARN7149 was the other compound for which we obtained indications on the possible mechanism of action. This compound was effective in the PLC assay. It was also effective in the Ca^2+^ mobilization assay irrespective of expression of a particular ITPR type. Finally, it was inactive in the caged IP_3_ assay (Fig. 4) thus ruling out ITPRs as the target. All these results place ARN7149 site of action on an early step of the GPCR-PLC-ITPR cascade. Lack of effect on other stimuli, involving other receptors, indicates that P2RY2 is the target of ARN7149, as also indicated by results with the AR‐C 118925XX antagonist (Fig. 5). Results obtained with the antagonist indicate that P2RY2, and not another purinergic receptor, is involved in ARN7149 activity. Potentiators of P2RY2 receptors could be potentially useful as therapeutic agents for a series of human diseases (29). In particular, they can be used topically to improve fluid and mucin secretion in dry eye syndrome (30, 31). A non-nucleotide agonist of P2RY2 attenuated isoprotenerol-induced cardiomyocyte hypertrophy (32). Potentiation of P2RY2 could also be useful to promote Ca^2+^-dependent Cl^−^ secretion in airway epithelia. In this respect, there is evidence of a tonic release of ATP by airway epithelia that is further enhanced by mechanical stress (33–35). Released ATP then acts in an autocrine way on epithelial cells activating TMEM16A through P2RY2. Therefore, ARN7149-like compounds could enhance Cl^−^ secretion and airway surface hydration, an effect that could be beneficial in CF and other chronic obstructive respiratory diseases. However, intracellular Ca^2+^ elevation could also promote mucus secretion. Future studies are needed to understand if potentiators of P2RY2 are effective in improving mucociliary clearance in the airways. In this respect, ARN7149 appears as a useful tool for such studies.

Regarding ARN4550, the third active compound found in our screening, we could not identify the precise site of action. This compound does not act on ITPRs since it was inactive in IP_3_ uncaging experiments. Also, it does not act on a specific GPCR, since it potentiated the Ca^2+^ mobilization by both UTP and SLIGR-NH2 stimuli. Therefore, ARN4550 may act on a step intermediate between GPCR and PLC, possibly a G protein, the PLC itself, or another related regulatory protein.

In conclusion, our study has used a functional screening assay to identify novel modulators of the Ca^2+^ signaling cascade. Such compounds will be important as mechanistic probes for scientific research purposes and as possible starting points in the development of therapeutic agents. Future studies are needed to further evaluate the selectivity of these modulators. This information is important to prevent off-target effects, which can be possibly minimized by modification of the chemical structure. Another aspect to consider, particularly relevant for therapeutic approaches, is the possibility of undesired effects due to activity of the compound on its primary target. In this respect, our compounds will be valuable to understand the role of their target in various physiological and pathological conditions.

## Materials and Methods

### Chemicals

ARN7149 (PubChem CID: 110084502), ARN4550 (PubChem CID: 46967215), and ARN11391 (PubChem CID: 46969526) were purchased from AKos GmbH, Germany (catalog number: AKOS030266631, AKOS030460511, AKOS030462755, respectively). Compounds were dissolved as 10 mM stock solutions in DMSO.

### Cell culture

Fischer rat thyroid (FRT) cells were cultured in a medium containing the Coon’s modification of Ham’s F12 plus 10% fetal bovine serum, 2 mM L-glutamine, 100 U/ml penicillin and 100 μg/ml streptomycin. Generation of FRT cells co-expressing the TMEM16A(*abc*) isoform and the halide-sensitive yellow fluorescent protein (HS-YFP) with the triple mutation H148Q/I152L/F46L was previously described (12). FRT cells were also separately transfected to generate a stable clone expressing the PH-PLCδ-GFP sensor.

HEK293 cells totally devoid of ITPR expression (HEK3XKO) or with selective expression of ITPR1 (HEKR1), ITPR2 (HEKR2), or ITPR3 (HEKR3), obtained by selective gene ablation, were purchased from Kerafast and cultured in a medium containing Dulbecco’s Modified Eagle Medium (DMEM, high glucose version) and Ham’s F12 (1:1 ratio) plus 10% fetal bovine serum, 2 mM L-glutamine, 100 U/ml penicillin and 100 µg/ml streptomycin. The same medium was also used to grow HEK293 cells stably transfected with tetracycline-inducible expression of wild type or mutant (R269W or T267M) ITPR1, kindly provided by Prof. S.R. Wayne Chen (University of Calgary). These cells were selected with 0.5 mg/ml hygromycin B and induced with 1 µg/ml tetracycline.

Human bronchial epithelial cells (HBECs) were collected and propagated as reported in a previous study (21). Briefly, HBECs were cultured in flasks in a home-made serum-free medium enriched with hormones and growth factors (21). After four to five passages, cells were seeded on Snapwell porous insert (cc3801, Corning Costar) at a density of 500,000/cm^2^. After 24 h from seeding, the basolateral medium was replaced with PneumaCult ALI (Stemcell Technologies) to promote mucociliary differentiation. The apical medium was instead removed to generate the air-liquid interface (ALI) condition. Epithelia were maintained for 2 to 3 weeks under ALI condition before experiments.

### HS-YFP assay and library screening

The HS-YFP assay was carried out with a FLUOstar Omega (BMG Labtech, Offenburg, Germany) microplate reader equipped with injection pumps and excitation/emission optical filters optimized for Enhanced Yellow Fluorescent Protein (EYFP) fluorescence (ET500/20x and ET535/30 m; Chroma Technology Corporation). FRT cells co-expressing TMEM16A and HS-YFP were plated at high density (50,000/well) in black wall/clear bottom 96-well microplates (cc3603, Corning). Each well in microplates was washed 3 times with 150 µl of phosphate-buffered saline (PBS). After washing, each well received 60 μl of PBS containing test compounds at 10 µM final concentration, Ani9 (10 µM), or vehicle. After incubation (20 min, 37°C), microplates were moved to the plate reader for HS-YFP assay. The microplate reader was programmed to process one well at a time by reading cell fluorescence every 200 ms for 14 s. At two seconds from start, 165 μl of a modified PBS containing 137 mM KI, instead of NaCl, plus UTP 0.25 µM were automatically added by the reader. K^+^ was used in the injected solution to prevent changes of membrane potential due to altered activity of Ca^2+^-activated K^+^ channels. The fluorescence trace in each well was corrected by background subtraction and then normalized for the initial fluorescence value measured before I^−^ addition (F0). The cumulative fluorescence quenching (CFQ) in the time interval between 3 and 13.8 s (representing integration of TMEM16A-dependent I^−^ influx) was quantified using a procedure compiled in Microsoft Excel according to the formula $\mathop \sum \limits_{i\ = \ 3}^{13.8} \Delta ( {Fi - F0} )$. HS-YFP assay was also done on HEK293 cells devoid of ITPR expression (HEK3XKO) after transient co-transfection with plasmids coding for TMEM16A(*abc*) and HS-YFP.

### Microplate reader-based Ca^2+^ assay

Experiments were carried out using the microplate reader described for the HS-YFP assay. FRT and HEK293 cells were cultured until confluence in 96-well microplates (cc3603, Corning). Cells were washed 2 times with PBS (150 μl/wash) and then incubated (1 h, 37°C) with 5 μM Fluo-4/AM (F23917, Thermo Fisher Scientific) in PBS containing 10 mM glucose, 0.5 mM sulfinpyrazone, and 1% fetal bovine serum. After loading, cells were washed 2 times with the PBS-glucose-sulfinpyrazone solution (150 μl/wash) and incubated (20 min, 37°C) with 60 μl of PBS-glucose-sulfinpyrazone solution plus compounds of interest at 10 µM final concentration or vehicle. Each well was assayed individually. Fluo-4 fluorescence (500 nm excitation, 535 nm emission) was detected every 200 ms for 16 to 40 s. Two seconds after the start of fluorescence recording, the reader injected 165 µl of a modified PBS containing 136 mM KCl instead of NaCl. This solution also contained 10 mM glucose, 0.5 mM sulfinpyrazone, and UTP or SLIGR-NH2 at a final concentration that depended on the specific cell type. For UTP we used: 0.25 µM for FRT; 1 µM for HEKR2; 5 µM for HEK3XKO, HEKR1, and HEKR3. For SLIGR-NH2 we used: 10 µM for HEKR1; 4 µM for HEKR2. These concentrations were chosen to elicit ∼20% of maximal effect based on UTP and SLIGR-NH2 dose-response relationships. The lower agonist concentrations used for HEKR2 cells, compared to HEKR1 cells, are consistent with the higher affinity of ITPR2 for IP_3_ (36). The Fluo-4 fluorescence increase was quantified by background subtraction and normalization for the initial fluorescence value measured before UTP addition.

### Intracellular Ca^2+^ imaging with ci-IP_3_/PM uncaging assay

FRT and HEK293 cells were cultured in µ-Plate96 microplates (Ibidi) at subconfluent condition. Cells were washed 2 times with PBS (150 μl/wash) and then incubated (45 min, 37°C) with 1 μM ci-IP_3_/PM (6210/10 U, Tocris) and 5 µM Fluo-4/AM (F23917, Thermo Fisher Scientific) in PBS containing 10 mM glucose, 0.5 mM sulfinpyrazone and 1% fetal bovine serum. After 45 min, cells were further incubated (1 h, 37°C) with a fresh PBS solution containing Fluo-4/AM, glucose, sulfinpyrazone, and serum, but no ci-IP_3_/PM. After the second loading step, cells were washed 2 times with PBS-glucose-sulfinpyrazone solution (150 μl/wash) and incubated (20 min, 37°C) with 125 μl of PBS-glucose-sulfinpyrazone solution also containing compounds of interest at 10 µM final concentration or vehicle. Each well was assayed individually with a system consisting of an inverted Olympus microscope equipped with a 40x oil immersion objective and a 530 nm emission filter, a high speed wavelength switcher (Lambda DG4, Sutter Instrument Co., Novato, CA, USA) carrying a 490 nm excitation filter, a Prime cmos camera (Photometrics, Tucson, AZ, USA), and the MetaFluor acquisition software (Molecular Devices, Sunnyvale, CA, USA). Time-lapse fluorescence microscopy experiments were carried out using the following conditions: 50 ms exposure time, 0.5 s interval, and 1.8 min total duration. At 50 s, illumination was switched for 250 ms to full lamp power (no excitation filter) to generate an intense light flash and induce ci-IP_3_/PM photolysis and hence IP_3_ uncaging. Analysis was performed with MetaFluor software, by measuring Fluo-4 fluorescence in manually selected single cell regions of interest (ROI). After background subtraction, the fluorescence trace for each ROI was normalized for the initial value. Eight cells were selected in each field to generate an average trace.

### PLC assay

FRT cells with stable expression of PLCδ-PH-GFP were cultured up to subconfluent condition in µ-Plate96 microplates (Ibidi). Each well was washed 3 times with PBS. Cells were then incubated (20 min, 37°C) with 125 μl of PBS containing compounds at 10 µM final concentration or vehicle. After incubation, the microplate was moved on the stage of an inverted microscope equipped with GFP excitation/emission filters, 40X oil immersion objective (Olympus, Segrate, Italy), a high speed wavelength switcher (Lambda DG4, Sutter Instrument Co., Novato, CA, USA), a Prime cmos camera (Photometrics, Tucson, AZ, USA), and the MetaMorph software (Molecular Devices, Sunnyvale, CA, USA). Time-lapse experiments were carried out with the following conditions: 50 ms exposure time, 20 frame/s rate, and 2.4 min total duration. After 20 s and 90 s, UTP 0.25 µM and 100 µM (final concentrations) was respectively added. Changes in cell fluorescence were quantified with MetaMorph software, by measuring GFP fluorescence cytoplasmic accumulation in manually selected regions of interest (ROI). After background subtraction, fluorescence values were normalized for the initial value.

### Short-circuit current recordings

Snapwell porous membrane supports (cc3801, Corning) carrying FRT or differentiated HBECs were mounted in an 8-channel Ussing-like system (EM-CSYS-8, Physiologic Instruments, San Diego, CA, USA). For FRT epithelia, the apical and basolateral compartments were filled with solutions of different Cl^−^ concentration to create a gradient (17). The low Cl^−^ solution in the apical compartment (5 ml) contained (in mM): 63 NaCl, 63 sodium gluconate, 0.38 KH_2_PO_4_, 2.13 K_2_HPO_4_, 2 CaCl_2_, 1 MgSO_4_, 10 glucose, and 20 Na-Hepes (pH adjusted to 7.3). The normal Cl^−^ solution in the basolateral compartment (5 ml) contained (in mM): 126 KCl, 0.38 KH_2_PO_4_, 2.13 K_2_HPO_4_, 1 CaCl_2_, 1 MgSO_4_, 10 glucose, and 20 Na-Hepes (pH adjusted to 7.3). Both compartments were continuously bubbled with air. Experiments were done at 37°C. For HBECs, the same (bicarbonate-buffered) solution was used in both compartments. The composition of this solution was (in mM): 126 NaCl, 0.38 KH_2_PO_4_, 2.13 K_2_HPO_4_, 1 MgSO_4_, 1 CaCl_2_, 10 glucose, and 24 NaHCO_3_. To achieve the desired pH (7.4), the solution in both compartments was continuously bubbled with 5% CO_2_ in air. Experiments were done at 37°C.

Transepithelial voltage was clamped at 0 mV with a VCC MC8 amplifier (Physiologic Instruments, San Diego, CA, USA) connected to the apical and basolateral compartments with voltage-sensing and current-passing Ag-AgCl electrodes immersed in agar bridges (2% agar, 1 M KCl). The offset between voltage-sensing electrodes and the fluid resistance were compensated before each set of experiments. The short-circuited current from each channel was recorded on a personal computer using the Acquire & Analize 2.3 software (Physiologic Instruments, San Diego, CA, USA).

### Patch-clamp recordings

Nuclear membrane patch-clamp currents were recorded in HEKR1 cells stably transfected with a plasmid encoding the human ITPR1 tagged with the Enhanced Yellow Fluorescent Protein (EYFP) kindly provided by Prof. Colin W. Taylor (University of Cambridge). For nuclei isolation, we used at least 1,000,000 HEKR1-ITPR1-EYFP cells plated in a 35 mm Petri dish following the protocol for patch clamp on the outer nuclear membrane (25, 26). Briefly, we used a Duall homogenizer 20 tube (Kimble Chase 885482–0020) and a tissue grind (Kimble 885480–0020). We applied 20 strokes to cells suspended in NIS-O solution (25). NIS-O solution consisted of 40 ml of sucrose buffer (in mM: 150 KCl, 250 sucrose, 1.4 β-mercaptoethanol, 10 Tris-HCl; pH adjusted to 7.3 with KOH) supplemented with one tablet of complete protease inhibitor cocktail (Roche) and 200 µM PMSF.

Borosilicate glass pipettes were pulled on a two-step vertical puller (Narishige) to a final resistance of around 15 to 20 MΩ, as measured in the working solution. The bath solution had the following composition (in mM): 140 KCl, 0.06 CaCl_2_, 0.5 EGTA, 10 K-Hepes (pH 7.3; free Ca^2+^ concentration: 70 nM). The pipette solution contained (in mM): 140 KCl, 0.5 EGTA, 0.46 CaCl_2_, 10 K-Hepes, 0.5 ATP (pH 7.3), 0.008 IP_3_. The pipette solution also contained 20 µM ARN11391 or DMSO.

For patch-clamp experiments, 50 µl of the suspension containing nuclei and intact cells were delivered to a 35 mm Petri dish, containing 2 ml of bath solution, and mounted on the stage of an inverted microscope. For each experiment, an isolated nucleus was chosen, using the criteria described by Mak and coll. (25, 26), to attempt giga-seal formation (on-nucleus patch-clamp configuration, equivalent to cell-attached patch-clamp).

During experiments, carried out with an EPC10 patch-clamp amplifier, the membrane capacitance was compensated. Experiments were performed at room temperature (20°C to 22°C). During recordings, the voltage pipette (Vp) was held at 0 mV with respect to the bath electrode and 5 s-long voltage steps to Vp = + 40 mV were applied with interval time of 1 s. Membrane currents were filtered (lowpass filter) at a frequency of 1 kHz and sampled at 10 kHz. Data were analyzed using the IGOR Pro software (WaveMetrics, Lake Oswego, OR, USA) implemented with software generated by Dr. Oscar Moran (Institute of Biophysics, CNR, Genova, Italy).

### Data visualization and statistical analysis

Data are shown as representative images/traces and as scatter dot plots plus mean ± standard deviation. Each dot in the plots reports the results of an independent experiment. To assess significant differences between groups of data, we applied ANOVA followed by Dunnett’s or Tukey’s post hoc tests as appropriate. Statistical analysis was done with the PRISM software (GraphPad). All graphs and figures were prepared with Igor Pro (WaveMetrics).

## Data Availability

All data and technical information are included in the manuscript.

## Notes

Present address: Alejandra Rodriguez-Gimeno, Department of Pharmacy and Pharmaceutical Technology and Physical Chemistry, University of Barcelona.
